# 527. A Descriptive, Retrospective Analysis of COVID-19 Antibody Therapy and its Effects on Morbidity and Mortality in Patients Receiving B-cell Depleting Therapies

**DOI:** 10.1093/ofid/ofad500.596

**Published:** 2023-11-27

**Authors:** G O R D A N A SIMEUNOVIC, Liam R Sullivan, Heather Brooks, Sonia K Gentile

**Affiliations:** Corewell Health/ Michigan State University, Grand Rapids, Michigan; Corewell Health (formerly Spectrum Health), Grand Rapids, Michigan; Corewell Health, Rockford, Michigan; Corewell Health, Michigan State University, Grand Rapids, Michigan

## Abstract

**Background:**

Patients receiving B-cell-depleting therapies (BCDT) are at an increased risk for severe COVID-19. Passive antibody therapy (PAT), including COVID-19 convalescent plasma (CCP) and monoclonal antibodies (MAB), is hypothesized to be an effective treatment in this population. However, real-world data on their effectiveness is limited.

**Methods:**

We conducted a retrospective chart review of patients who contracted COVID-19 within a year from their last BCDT treatment and later received PAT (Table 1). Response to treatment was assessed by 90-day COVID-related mortality and all-cause morbidity, defined through number of hospitalizations (Figure 1).

**Table 1.**
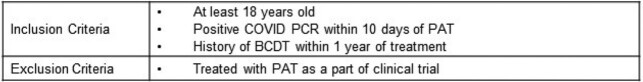
Inclusion and exclusion criteria.

**Figure 1.**
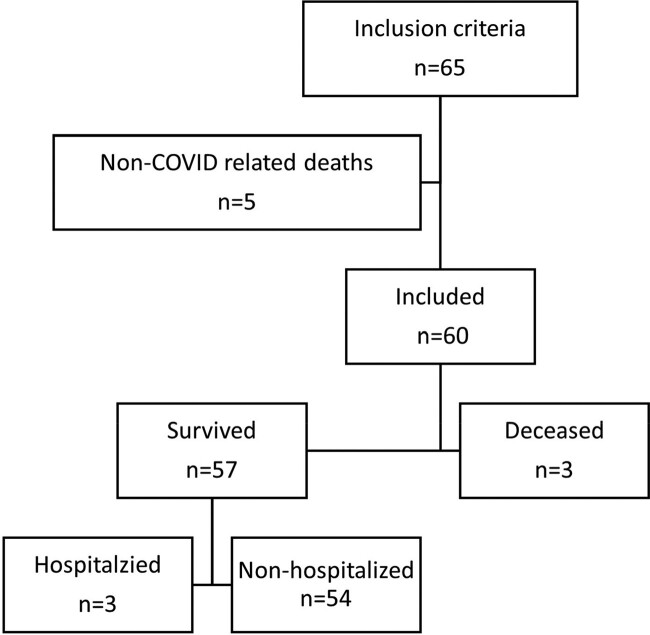
Patient flow.

Sixty-five patients met initial criteria. Five were excluded from analysis due to non-COVID related death within 90 days from COVID diagnosis. Cause of death was established in the chart and confirmed by review of two investigators.

**Results:**

From 60 included patients, the majority were Caucasians (97%), females (57%), and vaccinated (67%) (Table 2). Most patients received rituximab (53%) for treatment of a hematological malignancy (37%) or multiple sclerosis (37%) (Figure 2). Overall morbidity (3/39, 7.7%) and mortality (3/60, 5%) were low. All hospitalized and deceased patients were elderly Caucasian males receiving rituximab for underlying hematological malignancy. All deceased patients received inpatient treatment, 2 with CCP and one with MAB (Figure 3).

**Table 2.**
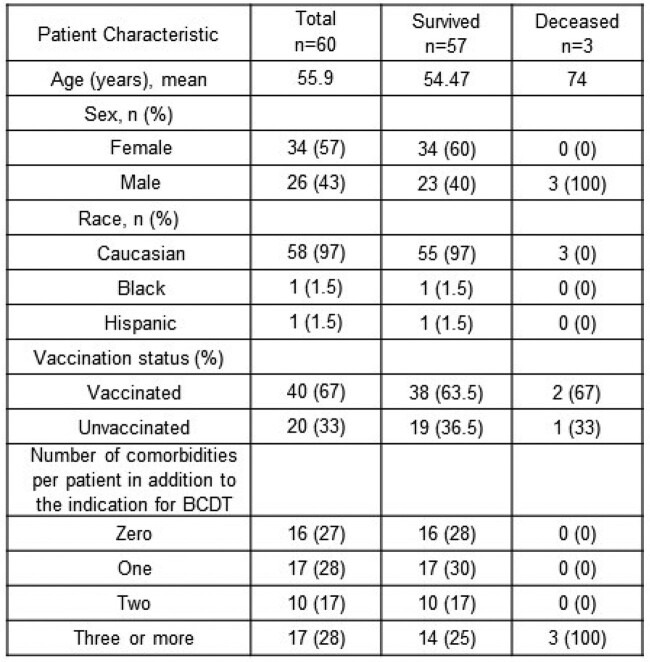
Basic characteristics of passive antibody therapy recipients.

Demographics, vaccination status and number of comorbidities excluding the condition which is the indication for B-cell depleting therapy.

**Figure 2.**
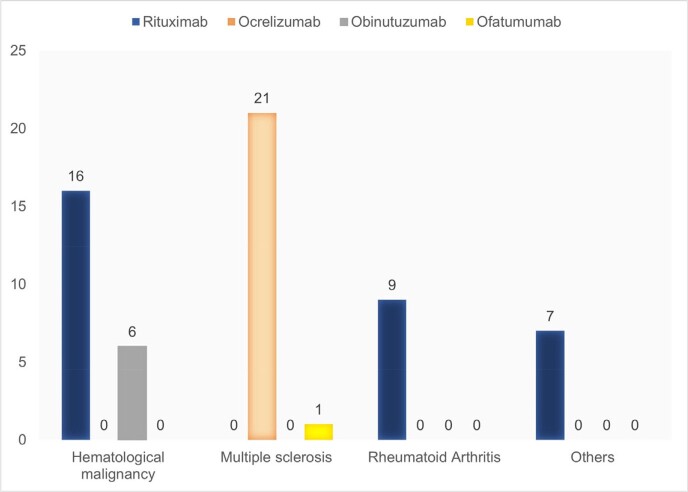
Type of B-cell depleting therapy and indication for use. Evaluated B-cell depleting therapies are rituximab (n=32), ocrelizumab (n=21), obinutuzumab (n=6), ofatumumab (n=1). Evaluated underlying indications for B-cell depleting therapy are hematological malignancy (n=22), multiple sclerosis (n=22); rheumatoid arthritis (n=9) and others (n=7). Others include scleroderma (n=1), systemic lupus (n=2), granulomatosis with polyangiitis (n=4).

**Figure 3:**
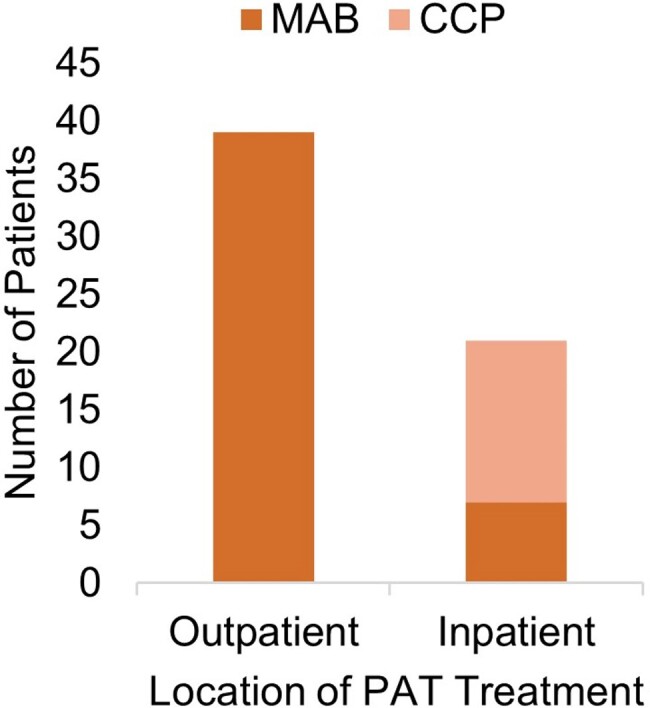
Treatment location and type of passive antibody therapy. All patients treated outpatient (39) received monoclonal antibodies. Of inpatients (21), 14 received COVID-19 convalescent plasma. Of patients treated as outpatient, 3 were hospitalized, with only 1 hospitalized for a COVID-related illness.

**Conclusion:**

COVID-19 patients undergoing BCDT and treated with PAT had low morbidity and mortality in our study, suggesting a possible benefit of PAT in this patient population. All deaths occurred in patients hospitalized at the time of treatment with PAT, implying advanced COVID-19 infection and highlighting the importance of early PAT administration. All deceased patients and 2/3 hospitalized patients were receiving rituximab, suggesting that rituximab may be a risk factor for severe COVID (Figure 4). All deceased and hospitalized patients had an underlying hematological malignancy, suggesting that the presence of hematologic malignancy may impact the outcome of COVID-19. In our study, elderly Caucasian males with multiple medical comorbidities and underlying hematological malignancy treated with BCDT, particularly rituximab, may have an increased risk for severe COVID-19. Early PAT administration may improve outcomes in this group of patients, and they should be prioritized for treatment when access to PAT is limited.

**Figure 4:**
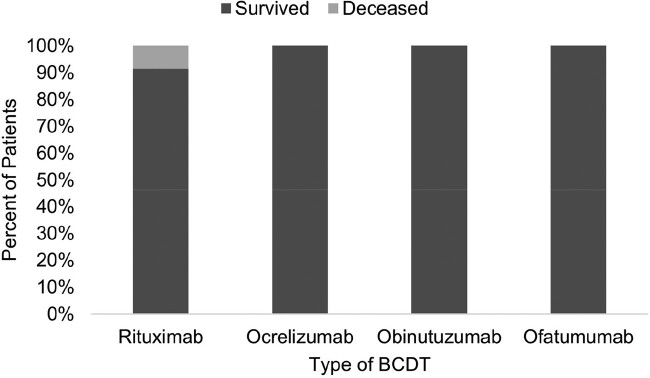
Type of B-cell depleting therapy and mortality.

All deaths were among patients being treated with rituximab for hematological malignancy.

**Disclosures:**

**All Authors**: No reported disclosures

